# Association of β-Blocker Use With Heart Failure Hospitalizations and Cardiovascular Disease Mortality Among Patients With Heart Failure With a Preserved Ejection Fraction: A Secondary Analysis of the TOPCAT Trial

**DOI:** 10.1001/jamanetworkopen.2019.16598

**Published:** 2019-12-04

**Authors:** Daniel N. Silverman, Timothy B. Plante, Margaret Infeld, Peter W. Callas, Stephen P. Juraschek, Geoff B. Dougherty, Markus Meyer

**Affiliations:** 1 Department of Medicine and Biostatistics Unit, Larner College of Medicine, University of Vermont, Burlington; 2 Department of Medicine, Beth Israel Deaconess Medical Center, Harvard Medical School, Boston, Massachusetts; 3 Department of Epidemiology, Johns Hopkins Bloomberg School of Public Health, Johns Hopkins University, Baltimore, Maryland

## Abstract

**Importance:**

β-Blockers are prescribed to most patients with heart failure (HF) with a preserved ejection fraction (HFpEF), but their effect on HFpEF remains unclear.

**Objective:**

To determine the association of β-blocker use with HF hospitalizations and cardiovascular disease (CVD) mortality, overall and in strata of patients with an ejection fraction (EF) of 50% or greater or less than 50%.

**Design, Setting, and Participants:**

For 1761 participants from North and South America enrolled in the multicenter, double-blinded Treatment of Preserved Cardiac Function Heart Failure with an Aldosterone Antagonist randomized clinical trial of spironolactone for patients with HFpEF between August 10, 2006, and January 31, 2012, the association of baseline β-blocker use with HF hospitalization and CVD mortality was analyzed using unadjusted and adjusted Cox proportional hazards regression models, overall and in strata of patients with an EF of 50% or greater or less than 50%. Participants had symptomatic HF with a left ventricular EF of 45% or greater, with enrollment based on either hospitalization attributed to decompensated HF in the prior year or elevated natriuretic peptide levels. Statistical analysis was performed from January 31 to May 2, 2019.

**Exposure:**

Use of β-blockers.

**Main Outcomes and Measures:**

Incident HF hospitalization and CVD mortality.

**Results:**

Among 1761 participants included in the analysis (879 women and 882 men; mean [SD] age, 71.5 [9.6] years), 1394 (79.2%) reported β-blocker use and 1567 (89.0%) had an EF of 50% or greater. Hospitalizations for HF occurred for 399 participants (22.7%), and CVD mortality occurred for 229 participants (13.0%). Use of β-blockers was associated with a higher risk of HF hospitalization among patients with HFpEF with an EF of 50% or greater (hazard ratio, 1.74 [95% CI, 1.28-2.37]; *P* < .001) but not among patients with an EF between 45% and 49% (hazard ratio, 0.68 [95% CI, 0.28-1.63]; *P* = .39). There was a significant interaction between β-blocker use and EF threshold for incident HF hospitalizations (*P* = .03). Use of β-blockers was not associated with a change in CVD mortality.

**Conclusions and Relevance:**

For patients with an EF of 50% or greater, β-blocker use was associated with an increased risk of HF hospitalizations but not CVD mortality. For patients with an EF between 45% and 49%, there was no such association.

## Key Points

**Question:**

Is there an association of β-blocker use with heart failure hospitalizations and cardiovascular disease mortality among patients with heart failure with a preserved ejection fraction?

**Findings:**

In this secondary analysis of the Treatment of Preserved Cardiac Function Heart Failure with an Aldosterone Antagonist randomized clinical trial of spironolactone for patients with heart failure with a preserved ejection fraction of 50% or greater, β-blocker use was associated with a higher risk of heart failure hospitalizations compared with patients not taking β-blockers. This association was not present among patients with an ejection fraction between 45% and 49%.

**Meaning:**

Prospective studies of the role β-blockers play in heart failure among patients with a preserved ejection fraction appears to be warranted to clarify the effectiveness of these drugs for patients with an ejection fraction of 50% or greater.

## Teaser

This secondary analysis of the TOPCAT randomized clinical trial examines the association of β-blocker use with heart failure (HF) hospitalizations and cardiovascular disease (CVD) mortality, overall and in strata of patients with an ejection fraction (EF) of 50% or greater or less than 50%.

## Introduction

Heart failure (HF) is a leading cause of hospitalizations and is associated with increased health care costs.^1,2^ More than half of the patients with HF have a preserved ejection fraction (HFpEF), defined as an ejection fraction (EF) of 50% or greater. Heart failure with a preserved EF continues to increase in prevalence and is associated with a high rate of hospitalization, yet, to our knowledge, evidenced-based therapies are lacking.^1,2,3,4^

β-Adrenergic receptor blockers (β-blockers) provide an unequivocal benefit in the treatment of chronic HF with a reduced EF (HFrEF), with a strong foundation of evidence to support their use.^5,6,7,8,9,10,11^ Most patients with HFpEF enrolled in contemporary clinical trials or in published cohorts also receive β-blockers, despite an uncertain benefit.^12,13,14,15,16,17^ A recent patient-based meta-analysis of 11 randomized β-blocker HF trials that enrolled patients with HFrEF also included a small number of patients with HFpEF.^18^ This analysis reinforced the benefit of β-blockers for patients with a reduced EF but did not demonstrate any benefit for patients with an EF greater than 50%.

To extend our understanding of the role β-blockers play in HFpEF, we performed an analysis of data from participants randomized in the Treatment of Preserved Cardiac Function Heart Failure with an Aldosterone Antagonist (TOPCAT) clinical trial. The primary focus of this analysis was to investigate the association of β-blocker use with HF hospitalizations among patients at different EF thresholds.^11^

## Methods

### TOPCAT Trial Design

The TOPCAT trial and its design have been previously described in detail, as have its main results.^19,20^ The trial was an international, multicenter, double-blinded, randomized clinical trial of the aldosterone antagonist spironolactone for patients with HFpEF, defined as symptomatic HF at the time of screening and within the preceding 12 months in patients with a left ventricular EF of 45% or greater. Enrollment took place between August 10, 2006, and January 31, 2012, with mean follow-up of 3.3 years, and was based on either a hospitalization attributed to decompensated HF in the preceding year or elevated brain natriuretic peptide (BNP) or N-terminal pro BNP (NT-proBNP) levels (BNP level ≥100 pg/mL [to convert to nanograms per liter, multiply by 1.0] or NT-proBNP level ≥360 pg/mL) within 60 days of screening.^19,20^ The initial comprehensive baseline visit included a detailed medical history of comorbidities, social history, risk factor documentation, medication survey, quality of life questionnaire, and assessment of physical activity and medications. Not all follow-up visits included a medication inventory.^20^ The University of Vermont Institutional Review Board deemed this research to be exempt from review as it was a retrospective analysis performed on a deidentified data set. Enrolled patients provided written informed consent. This study followed the Consolidated Standards of Reporting Trials (CONSORT) reporting guideline.

### Analyzed Population

We used the deidentified TOPCAT database from the National Heart, Lung, and Blood Institute Biologic Specimen and Data Repositories Information Coordinating Center. Because of reported trial quality concerns in some regions, we analyzed the data only from South America and North America.^21,22,23^ Patients without a baseline EF and those without a recorded baseline visit were also excluded. We used baseline and follow-up medication logs to chart β-blocker use.

### Statistical Analysis

Statistical analysis was performed from January 31 to May 2, 2019. Baseline characteristics were tabulated by use of a β-blocker vs no use of a β-blocker. β-Blocker use itself was defined as the receipt of any β-blocker at the baseline visit. To assess the association between β-blockers and incident HF hospitalizations, hazard ratios were calculated comparing baseline β-blocker use vs no β-blocker use and stratified by an EF of 50% or greater or less than 50%. This threshold was chosen because it allows for a clinically meaningful separation between HF phenotypes as recommended by current guidelines.^5,6^ In a minimally adjusted covariate model, we corrected for age, sex, race/ethnicity, and treatment assignment (spironolactone or placebo). In the fully adjusted model, baseline myocardial infarction, atrial fibrillation, chronic obstructive pulmonary disease, asthma, and hypertension were added to the minimally adjusted model. This analysis was repeated for cardiovascular disease (CVD) mortality. Schoenfeld residuals were examined to confirm no violation of the assumptions of the Cox proportional hazards regression model.

The association between level of baseline EF and relative hazard of HF hospitalization for those receiving β-blockers or not in the fully adjusted model was presented using restricted cubic spline models with 95% CIs relative to the median. Knots were not prespecified and were chosen using the Harrell method.^24^ The distribution of the baseline EF in both groups by HF hospitalizations was visualized using kernel density plots.

We performed a sensitivity analysis among the participants whose β-blocker status was constant from baseline through each follow-up visit, censoring at the end of follow-up or at the first HF hospitalization. In this population with consistent documentation of β-blocker or no β-blocker use, we calculated relative hazards using the unadjusted, minimally adjusted, and fully adjusted models for HF hospitalizations overall and in the prespecified EF strata. In a second sensitivity analysis among the participants whose β-blocker status was constant, propensity scores were created using 30 baseline demographic, anthropometric, medication, and medical history patient characteristics. Up to 3 patients receiving β-blockers were matched to each patient not receiving a β-blocker. Heart failure hospitalization was analyzed for the propensity score–matched data using stratified Cox proportional hazards regression, with the matched sets as the stratifying variable.

To provide some pathomechanistical insights, we compared BNP and NT-proBNP levels at baseline by β-blocker status overall and stratified by an EF of 50%. We compared log-transformed baseline levels of BNP and NT-proBNP using unadjusted 2-tailed *t* tests. We visualized distributions of the quartiles of the BNPs and NT-proBNPs and the midrange of each quartile among those with an EF of 50% or greater using stacked bar graphs.

The analyses were performed with Stata MP, version 15.1 (StataCorp) and the SAS, version 9.4 procedure PSMATCH (SAS Institute Inc) for the propensity score matching. We considered a 2-tailed *P* < .05 to be statistically significant.

## Results

### Study Population

**Figure 1.**
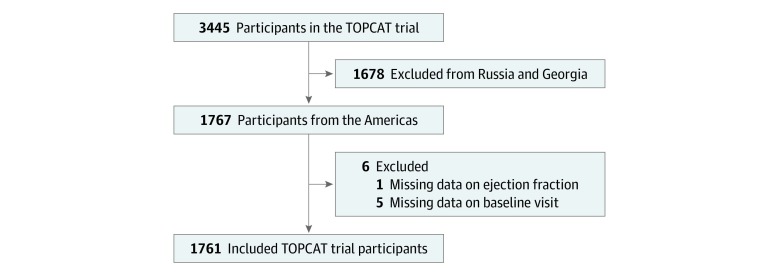
Flow Diagram of Trial Participants Flow diagram of patient inclusions and exclusions leading to the analyzed population. TOPCAT indicates Treatment of Preserved Cardiac Function Heart Failure with an Aldosterone Antagonist.

Among the 1767 TOPCAT participants from North America and South America, the mean (SD) age was 71.5 (9.6) years; 879 participants were women and 882 were men, and 1378 participants were white. Six participants were excluded because of missing EF data or missing baseline visit data (Figure 1). The final analytic population was 1761. Median follow-up was 2.4 years (interquartile range, 1.4-3.9 years).

A total of 1394 participants (79.2%) were receiving β-blockers at baseline and 1567 (89.0%) had an EF of 50% or greater (Table 1). Irrespective of β-blocker use, most patients had preexisting hypertension. The prevalence of atrial fibrillation was 42.6% (594 of 1393) among those receiving a β-blocker and 40.7% (149 of 366) among those not receiving a β-blocker, and the prevalence of prior myocardial infarction was 22.1% (308 of 1395) among those receiving a β-blocker and 13.7% (46 of 336) among those not receiving a β-blocker. The proportion of patients with an advanced functional impairment (New York Heart Association class ≥3) was almost identical between groups.

**Table 1.** Baseline Characteristics of Participants^a^

Characteristic	No β-Blocker (n = 367 [20.8%])	β-Blocker (n = 1394 [79.2%])
Spironolactone	187 (51.0)	696 (49.9)
Age, mean (SD), y	72.4 (10.2)	71.3 (9.5)
Female sex	207 (56.4)	672 (48.2)
Race/ethnicity		
White	291 (79.3)	1087 (78.0)
Black	59 (16.1)	243 (17.4)
Asian	7 (1.9)	12 (0.9)
Other	11 (3.0)	59 (4.2)
Hispanic	100 (27.2)	213 (15.3)
Anthropometric data, mean (SD)		
Heart rate, beats per min	71 (13)	69 (11)
BP, mm Hg		
Systolic	129 (16)	127 (16)
Diastolic	73 (12)	71 (11)
BMI	34 (9)	34 (8)
EF or heart failure		
EF, mean (SD)	59.2 (7.7)	57.9 (7.8)
EF ≥50%	337 (91.8)	1229 (88.2)
EF ≥60%	201 (54.8)	654 (46.9)
NYHA class ≥3	124/364 (34.1)	494/1393 (35.5)
Medical history		
Myocardial infarction	50 (13.6)	308/1393 (22.1)
Hypertension	312 (85.0)	1273/1393 (91.4)
Atrial fibrillation	149 (40.6)	594/1393 (42.6)
Medications		
ACEI, ARB, aliskiren	296 (80.7)	1099 (78.8)
Diuretic	315 (85.8)	1258 (90.2)
Thiazide	106 (28.9)	341 (24.5)
Loop diuretic	257 (70.0)	1128 (80.9)
Calcium channel blocker	166 (45.2)	516 (37.0)

Abbreviations: ACEI, angiotensin converting enzyme inhibitor; ARB, angiotensin receptor blocker; BMI, body mass index (calculated as weight in kilograms divided by height in meters squared); BP, blood pressure; EF, ejection fraction; NYHA, New York Heart Association.

^a^ Data are presented as number (percentage) of patients unless otherwise indicated.

### HF Hospitalizations and CVD Mortality

**Figure 2.**
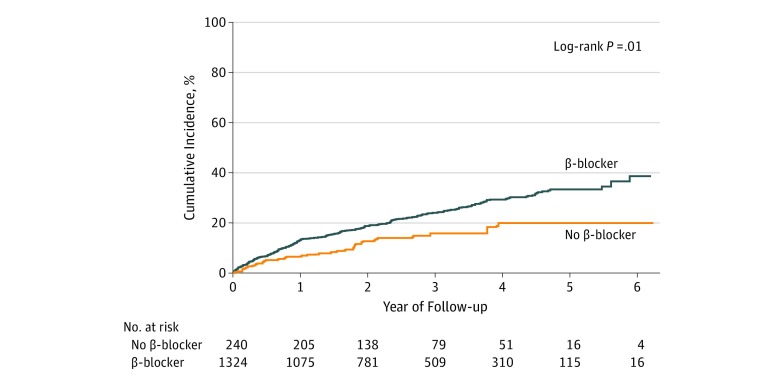
Cumulative Incidence for Heart Failure Hospitalizations by β-Blocker Use Among Patients With an Ejection Fraction of 50% or Greater Kaplan-Meier plots for heart failure hospitalizations by β-blocker use at baseline stratified by an ejection fraction of 50% or greater.

**Figure 3.**
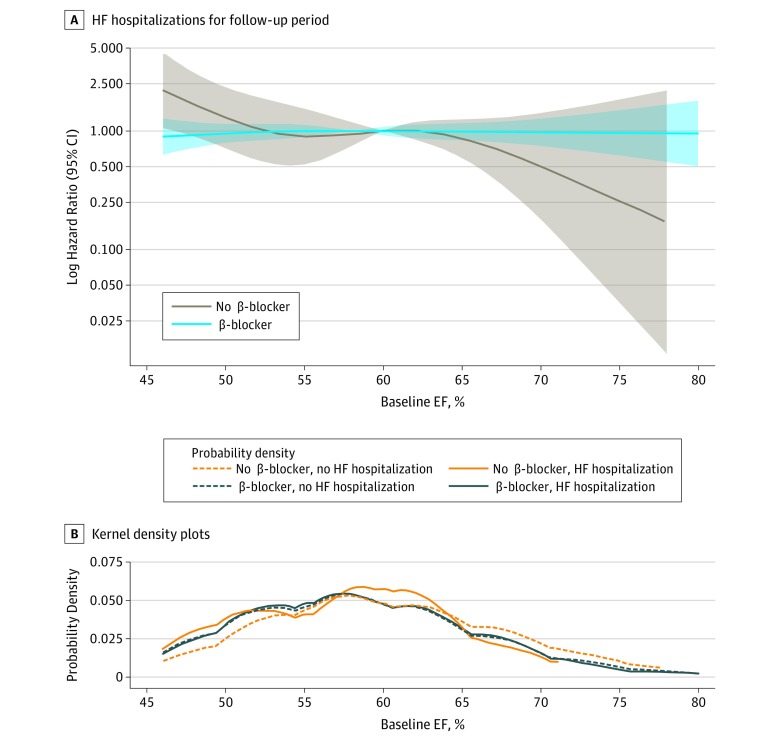
Restricted Cubic Splines and Kernel Density Plot Relating Hazard Ratios for Heart Failure (HF) Hospitalization and Ejection Fraction (EF) A, Hazard ratios for incident HF hospitalizations for the follow-up period, according to baseline EF using restricted cubic spline models, adjusted for age, sex, race/ethnicity, treatment assignment, prior myocardial infarction, atrial fibrillation, chronic obstructive pulmonary disease, asthma, and hypertension. The shaded areas represent the 95% CIs. The logarithmic scale on the y-axis indicates hazard ratios for HF hospitalization, where values greater than 1 indicate greater rate of HF hospitalizations and values less than 1 indicate fewer HF hospitalizations are related to an EF on the x-axis. The models were expressed relative to the median EF. Four knots were specified using the Harrell method and were not prespecified.^24^ Knots were 43.0%, 53.0%, 59.0%, and 71.7% for β-blocker and 47.0%, 57.0%, 62.0%, and 72.0% for no β-blocker. The plots were truncated at 0.5% and 99.5% of baseline EF. B, Kernel density plots demonstrating the distribution of baseline EFs.

Overall, 399 patients (22.7%) underwent hospitalization for HF (β-blocker group, 344 of 1394 [cumulative incidence, 24.7%; unadjusted incidence rate, 96 per 1000 person-years; 95% CI, 86-106]; no β-blocker group, 55 of 367 [cumulative incidence, 15.0%; unadjusted incidence rate, 56 per 1000 person-years; 95% CI, 43-73]). In the fully adjusted model, there was a higher incidence of HF hospitalizations among patients with an EF of 50% or greater who were receiving β-blockers (Table 2) compared with those not receiving β-blockers (hazard ratio, 1.74 [95% CI, 1.28-2.37]; *P* < .001) (Figure 2). There was a significant interaction between an EF threshold, β-blocker use, and incident HF hospitalization (*P* = .03); higher EFs were associated with an increased risk for HF hospitalizations among patients receiving β-blockers (Figure 3).^24^ Patients with the highest EFs who did not receive β-blockers were least likely to be admitted for HF. There was a nonsignificant trend toward a lower incidence of HF hospitalizations among patients with an EF between 45% and 49% who were receiving β-blockers (hazard ratio, 0.68 [95% CI, 0.28-1.63]; *P* = .39). Cardiovascular disease mortality occurred among 229 participants (13.0%); β-blockers were not significantly associated with a change in CVD mortality (eTable 6 and eFigure 1 in the Supplement).

**Table 2.** Hazard Ratios for Heart Failure Hospitalizations of Patients Receiving β-Blockers, by Ejection Fraction

Ejection Fraction	Unadjusted Hazard Ratio	Adjusted Hazard Ratio	
		Minimally^a^	Fully^b^
All	1.71 (1.28-2.27)	1.61 (1.21-2.14)	1.61 (1.20-2.15)
<50%	0.73 (0.32-1.65)	0.69 (0.29-1.64)	0.68 (0.28-1.63)
≥50%	1.86 (1.37-2.52)	1.74 (1.28-2.36)	1.74 (1.28-2.37)
≥55%	2.06 (1.45-2.92)	1.90 (1.33-2.70)	1.90 (1.33-2.71)
≥60%	2.03 (1.34-3.08)	1.84 (1.21-2.81)	1.80 (1.18-2.75)
≥65%	2.92 (1.46-5.82)	2.72 (1.35-5.47)	2.65 (1.31-5.36)

^a^ Adjusted for age, sex, race/ethnicity, and treatment assignment.

^b^ Minimally adjusted model plus prior myocardial infarction, atrial fibrillation, chronic obstructive pulmonary disease, asthma, and hypertension.

The sensitivity analysis that considered HF hospitalizations among patients who either continued receiving a β-blocker or who never received a β-blocker through the end of follow-up or first HF hospitalization confirmed the principal findings (eTable 1 in the Supplement). In the fully adjusted model, patients with an EF of 50% or greater receiving β-blockers had a higher relative hazard of HF hospitalization compared with those not receiving β-blockers (hazard ratio, 1.76 [95% CI, 1.22-2.53]; *P* = .007). In a second sensitivity analysis of propensity score–matched cohorts, β-blocker use was also associated with more HF hospitalizations among patients with EFs of 50% or greater (eTable 2 and eTable 3 in the Supplement). This analysis also confirmed that patients receiving β-blockers who had higher EFs had an associated risk of being hospitalized for HF.

### BNP and NT-proBNP Levels

β-Blocker use was associated with higher NT-proBNP and BNP levels in patients with an EF of 50% or greater (eTable 5 and eFigure 2 in the Supplement). This was not the case for patients with an EF between 45% and 49%.

## Discussion

To date, the efficacy of β-blockers for patients with HFpEF is unknown. This post hoc analysis of the TOPCAT trial of spironolactone for patients with HFpEF suggests an association between β-blocker use and incident HF hospitalizations for patients with an EF of 50% or greater and an incremental positive association between β-blocker use and the risk for HF hospitalization at higher EF thresholds. There was no significant association between β-blocker use and CVD mortality.

### β-Blocker Use for Patients With HFpEF

The TOPCAT trial corroborates the finding that most patients in contemporary HFpEF cohorts are treated with β-blockers. A total of 79.2% of the patients in the North American and South America cohort of this trial received β-blockers at baseline. This high prevalence of β-blocker use is similar to other HFpEF studies, as shown in eTable 4 in the Supplement .^12,13,14,15,16^ Although evidence for the benefits of β-blocker use for patients with HFpEF is lacking, this high rate of use is most likely explained by an assumption that β-blockers are efficacious for treating common comorbidities such as hypertension, coronary artery disease, and atrial fibrillation.

### Benefits of β-Blockers for HF

Extensive, high-quality evidence supports the use of β-blockers for patients with HFrEF.^5,6,9,10,11,25,26^ In addition, several recent analyses have investigated whether guideline-directed HF therapies have a utility for patients with EFs between 40% and 49%, an entity termed *HF with midrange EF*.^27,28,29^ A recent individual patient-level meta-analysis of 11 major HFrEF trials investigating the effects of β-blockers at different ranges of EF identified a reduction in CVD mortality among patients with an EF between 40% and 49%.^18^ Analogous to our findings, the same meta-analysis suggested that patients with an EF of 50% or greater did not see any benefits from being randomized to receive β-blockers. However, only 244 patients fell into this category.

### HFpEF Trials of β-Blockers

Our observations contrast with prior randomized β-blocker trials, which did not report increased HF hospitalizations among patients with HFpEF. However, there are several points to consider. First, to our knowledge, only 2 randomized clinical outcome trials have been performed that studied β-blockers in patients with HFpEF. The larger SENIORS trial (Randomized Trial to Determine the Effect of Nebivolol on Mortality and Cardiovascular Hospital Admission in Elderly Patients With Heart Failure) considered an EF greater than 35% to define HFpEF and analyzed 752 participants with an EF in this range, among whom approximately half had an EF between 35% and 50%.^30^ In addition, HF hospitalizations were not specifically recorded. The open-label Japanese Diastolic Heart Failure (J-DHF) trial of carvedilol (mean dose, 8.5 mg/d) randomized 245 patients with an EF greater than 40%.^31^ It appears possible that assessing HF hospitalizations in mixed populations with a reduced and normal EF may result in an overall neutral or even beneficial effect associated with β-blockers.^28,29^ Furthermore, patients with a history of HF and a recovered EF have recently been shown to gain sustained benefits from guideline-directed HFrEF therapies, which include β-blockers.^32^ Their inclusion in HFpEF cohorts—although likely small in numbers—could also be associated with beneficial HF outcomes.

### Possible Pathophysiological Mechanisms

Evidence that may help explain these findings comes from related patient populations with preserved EFs. Specifically, the LIFE (Losartan Intervention For Endpoint Reduction in Hypertension) trial^33^ and contemporary randomized myocardial infarction trials^34,35,36^ have raised concerns that β-blocker use is associated with adverse cardiovascular outcomes, including an increased risk of developing HF. Mechanistically, this risk was explained by an increase in central blood pressure by reflected pressure waves.^35^ In addition, prolonged diastolic filling increases ventricular volumes and pressures, increasing the ventricular load.^37,38^ These mechanisms combine to increase myocardial wall stress, which may explain why, in historical hypertension trials, BNP and NT-proBNP levels were found to be elevated in patients receiving β-blockers.^39^

Similarly, in the TOPCAT trial, β-blocker use was associated with higher levels of circulating BNPs and NT-proBNPs in patients with a normal EF. The same was seen in the ELANDD (Effects of the Long-term Administration of Nebivolol on the Clinical Symptoms, Exercise Capacity, and Left Ventricular Function of Patients With Diastolic Dysfunction) study, which assessed the effect of nebivolol on clinical symptoms and exercise capacity in patients with diastolic dysfunction, and in the CIBIS-ELD (Titration to Target Dose of Bisoprolol vs Carvedilol in Elderly Patients With Heart Failure) study, which evaluated the tolerability of β-blocker up-titration in patients with HFpEF.^40,41^ In the SWEDIC (Swedish Doppler-Echocardiographic) study of patients with HFpEF randomized to receive carvedilol or placebo, BNP levels also increased, and the authors noted an unexpected worsening in HF in the patients treated with carvedilol.^42^ As has been recently demonstrated, β-blocker cessation for patients with stable HFpEF is safe and leads to marked reductions in NTpro-BNP levels.^43^

Although incremental increases in BNP and NT-proBNP levels have been associated with worse outcomes for patients with HFpEF—including in another secondary analysis of the TOPCAT trial^44^—it is not clear whether this predictive capacity is preserved if indeed modified by β-blockers. However, in contrast with the observations of patients with HFpEF, BNP and NT-proBNP levels are markedly lowered by sustained β-blocker use among patients with HFrEF.^5,45^

### Limitations

Our examination has several limitations. Participants in the TOPCAT trial were randomized to receive spironolactone and not β-blockers. Our adjustments may not sufficiently correct for all confounding variables, and some confounders may be unidentified. We also cannot account for both duration and intensity of β-blocker exposure. This secondary analysis can be viewed only as explorative and hypothesis generating because it does not establish cause and effect.

## Conclusions

These results demonstrate that β-blocker use in the TOPCAT trial cohort was associated with a higher risk for incident HF hospitalization among patients with an EF of 50% or greater, without an associated change in CVD mortality. Future studies are needed to prospectively assess the effects of β-blockers in HF populations with a normal EF.

## Notes

Supplement. **eTable 1.** Sensitivity Analysis, Hazard Ratios for Heart Failure Hospitalizations by Ejection Fraction Among Those Not Changing Baseline Beta-Blocker Status During Follow-up **eTable 2.** Sensitivity Analysis, Hazard Ratios for Heart Failure Hospitalizations by Ejection Fraction Among Those Not Changing Baseline Beta-Blocker Status During Follow-up Using the Propensity Score Matched Cohort **eTable 3.** Baseline Characteristics of Propensity Score Matched Patients That Did Not Change Baseline Beta-Blocker Status During Follow-up **eTable 4.** Beta-blocker Utilization in Contemporary HFpEF Cohorts **eTable 5.** Median and Interquartile Ranges for BNP and NT-proBNP Levels **eTable 6.** Hazard Ratios for Cardiovascular Disease Mortality by Ejection Fraction **eFigure 1.** Cumulative Incidence Plot for Cardiovascular Disease Mortality by Beta-Blocker Use in Patients With an EF≥50% **eFigure 2.** Quartile Ranges of BNP and NT-proBNP Levels by Beta-Blocker Use
