# Supplementary material for: Association of β-Blocker Use With Heart Failure Hospitalizations and Cardiovascular Disease Mortality Among Patients With Heart Failure With a Preserved Ejection Fraction: A Secondary Analysis of the TOPCAT Trial
Source: JAMA Netw Open. 2019 Dec 4;2(12):e1916598. doi: 10.1001/jamanetworkopen.2019.16598 (PMC6902757; doi:10.1001/jamanetworkopen.2019.16598)
Supplement: Supplement. — eTable 1. Sensitivity Analysis, Hazard Ratios for Heart Failure Hospitalizations by Ejection Fraction Among Those Not Changing Baseline Beta-Blocker Status During Follow-up eTable 2. Sensitivity Analysis, Hazard Ratios for Heart Failure Hospitalizations by Ejection Fraction Among Those Not Changing Baseline Beta-Blocker Status During Follow-up Using the Propensity Score Matched Cohort eTable 3. Baseline Characteristics of Propensity Score Matched Patients That Did Not Change Baseline Beta-Blocker Status During Follow-up eTable 4. Beta-blocker Utilization in Contemporary HFpEF Cohorts eTable 5. Median and Interquartile Ranges for BNP and NT-proBNP Levels eTable 6. Hazard Ratios for Cardiovascular Disease Mortality by Ejection Fraction eFigure 1. Cumulative Incidence Plot for Cardiovascular Disease Mortality by Beta-Blocker Use in Patients With an EF≥50% eFigure 2. Quartile Ranges of BNP and NT-proBNP Levels by Beta-Blocker Use [file jamanetwopen-2-e1916598-s001.pdf]

Supplementary Online Content

Silverman DN, Plante TB, Infeld M, et al. Association of β-blocker use with heart failure hospitalizations and cardiovascular disease mortality among patients with heart failure with a preserved ejection fraction: a secondary analysis of the TOPCAT trial. *JAMA Netw Open.* 2019;2(12):e1916598. doi:10.1001/jamanetworkopen.2019.16598

eTable 1. Sensitivity Analysis, Hazard Ratios for Heart Failure Hospitalizations by Ejection Fraction Among Those Not Changing Baseline Beta-Blocker Status During Follow-up
eTable 2. Sensitivity Analysis, Hazard Ratios for Heart Failure Hospitalizations by Ejection Fraction Among Those Not Changing Baseline Beta-Blocker Status During Follow-up Using the Propensity Score Matched Cohort
eTable 3. Baseline Characteristics of Propensity Score Matched Patients That Did Not Change Baseline Beta-Blocker Status During Follow-up
eTable 4. Beta-blocker Utilization in Contemporary HFpEF Cohorts
eTable 5. Median and Interquartile Ranges for BNP and NT-proBNP Levels
eTable 6. Hazard Ratios for Cardiovascular Disease Mortality by Ejection Fraction
eFigure 1. Cumulative Incidence Plot for Cardiovascular Disease Mortality by Beta-Blocker Use in Patients With an EF ≥50%
eFigure 2. Quartile Ranges of BNP and NT-proBNP Levels by Beta-Blocker Use

This supplementary material has been provided by the authors to give readers additional information about their work.

© 2019 Silverman DN et al. *JAMA Network Open.*

eTable 1. Sensitivity Analysis, Hazard Ratios for Heart Failure Hospitalizations by Ejection Fraction Among Those Not Changing Baseline Beta-Blocker Status During Follow-up*

	**Unadjusted**	**Minimally Adjusted****	**Fully adjusted*****
**All EF**	1.66 (1.20, 2.30)	1.54 (1.11, 2.15)	1.55 (1.11, 2.17)
**EF <50%**	0.53 (0.23, 1.21)	0.51 (0.22, 1.18)	0.49 (0.21, 1.17)
**EF ≥50%**	1.90 (1.33, 2.71)	1.74 (1.21, 2.49)	1.76 (1.22, 2.53)
**EF ≥55%**	2.24 (1.47, 3.42)	2.04 (1.32, 3.12)	2.08 (1.35, 3.21)
**EF ≥60%**	2.29 (1.38, 3.78)	2.07 (1.24, 3.45)	2.06 (1.23, 3.47)
**EF ≥65%**	3.09 (1.34, 7.11)	2.83 (1.20, 6.64)	2.68 (1.13, 6.34)

*This analysis excludes the 284 participants who did not receive beta-blockers at baseline who had documented beta-blocker use during medication reconciliation at any follow-up visit (n=103) and those who received beta-blockers at baseline who had follow-up visits at which medication reconciliation documented no beta-blocker use (n=181). We censored at the time of first heart failure hospitalization for these comparisons. The interaction between an EF threshold of 50% and beta-blocker use and incident HF hospitalizations in the fully adjusted model achieved statistical significance (*p*=0.007).

*Adjusted for age, sex, race/ethnicity, and treatment assignment
** Minimally adjusted model plus prior myocardial infarction, atrial fibrillation, chronic obstructive pulmonary disease, asthma, and hypertension

© 2019 Silverman DN et al. *JAMA Network Open.*

eTable 2. Sensitivity Analysis, Hazard Ratios for Heart Failure Hospitalizations by Ejection Fraction Among Those Not Changing Baseline Beta-Blocker Status During Follow-up Using the Propensity Score Matched Cohort

	**Hazard Ratios**
**All EF**	1.36 (0.94, 1.97)
**EF ≥50%**	1.51 (0.99, 2.29)
**EF ≥55%**	1.71 (1.03, 2.83)
**EF ≥60%**	2.20 (1.06, 4.57)
**EF ≥65%**	2.66 (0.71, 9.93)

This analysis excludes participants who did not receive beta-blockers at baseline who had documented beta-blocker use during medication reconciliation at any follow-up visit and those who received beta-blockers at baseline who had follow-up visits at which medication reconciliation documented no beta-blocker use. We censored at the time of first heart failure hospitalization for these comparisons. The interaction between EF and beta-blocker use and incident HF hospitalizations was statistically significant (p=0.046).The hazard ratios for patients with an EF 45-49 could not be estimated due to the small number of matched sets.

© 2019 Silverman DN et al. *JAMA Network Open.*

eTable 3. Baseline Characteristics of Propensity Score Matched Patients That Did Not Change Baseline Beta-Blocker Status During Follow-up

		Beta Blocker		
		No	Yes	P value*
**N**		260	640	
**Treatment**	N (%) spironolactone	133 (51%)	323 (50%)	0.84
**Age**	Mean (SD) years	73 (10)	73 (10)	0.92
**Sex**	N (%) female	148 (57%)	352 (55%)	0.97
**Race**	N (%) white non-hispanic	145 (56%)	371 (58%)	0.88
**NYHA class ≥3**	N (%)	90 (35%)	222 (35%)	0.73
**Height**	Mean (SD) cm	166 (11)	165 (11)	0.47
**Weight**	Mean (SD) kg	92 (26)	92 (25)	0.78
**BMI**	Mean (SD)	33.5 (9.2)	33.5 (8.1)	0.93
**Waist**	Mean (SD) cm	110 (19)	108 (17)	0.15
**HR**	Mean (SD) bpm	71 (12)	70 (11)	0.90
**SBP**	Mean (SD) mmHg	129 (16)	128 (16)	0.89
**DBP**	Mean (SD) mmHg	73 (13)	72 (11)	0.78
**Baseline Medications/ Natriuretic Peptides**				
**ACE/ARB/Aliskirin**	N (%)	205 (79%)	486 (76%)	0.27
**Statin**	N (%)	123 (47%)	347 (54%)	0.95
**Other lipid medication**	N (%)	18 (7%)	47 (7%)	0.72
**Aspirin**	N (%)	118 (45%)	316 (49%)	0.98
**Other antiplatelet agent**	N (%)	20 (8%)	52 (8%)	0.74
**Anticoagulation**	N (%)	90 (35%)	244 (38%)	0.61
**Thiazide diuretic**	N (%)	76 (29%)	179 (28%)	0.99
**Loop diuretic**	N (%)	182(70%)	487 (76%)	0.51
**Other K sparing [illegible]**	N (%)	1 (<1%)	4 (1%)	0.44
**CAI diuretic**	N (%)	0 (0%)	1 (<1%)	0.48
**Any diuretic**	N (%)	225 (87%)	569 (89%)	0.80
**Ca channel blocker**	N (%)	119 (46%)	270 (42%)	0.84
**Digoxin**	N (%)	32 (12%)	79 (12%)	0.73
**BNP**	Mean (SD)	307 (226)	407 (486)	0.10
**NT-proBNP**	Mean (SD)	1787 (2296)	1807 (2159)	0.77
**BNP quartile**	N(%) 1	17 (19%)	61 (23%)	0.13
	N(%) 2	32 (36%)	62 (24%)	
	N(%) 3	23 (26%)	75 (29%)	
	N(%) 4	18 (20%)	65 (25%)	
**NT-proBNP quartile**	N(%) 1	10 (19%)	25 (17%)	0.31
	N(%) 2	11 (21%)	39 (27%)	
	N(%) 3	18 (34%)	27 (19%)	
	N(%) 4	14 (26%)	54 (37%)	

© 2019 Silverman DN et al. *JAMA Network Open.*

**Past Medical History**				
**MI**	N (%)	27 (10%)	80 (13%)	0.83
**Angina**	N (%)	44 (17%)	124 (19%)	0.99
**Stroke**	N (%)	26 (10%)	48 (8%)	0.19
**COPD**	N (%)	53 (20%)	120 (19%)	0.94
**Asthma**	N (%)	37 (14%)	85 (13%)	0.90
**HTN**	N (%)	220 (85%)	560 (88%)	0.87
**PAD**	N (%)	23 (9%)	52 (8%)	0.58
**Dyslipidemia**	N (%)	152 (58%)	405 (63%)	0.96
**Atrial fibrillation**	N (%)	100 (38%)	278 (43%)	0.56
**Thyroid**	N (%)	50 (19%)	124 (19%)	0.86
**Diabetes mellitus**	N (%)	96 (37%)	250 (39%)	0.92
**Smoking**	N (%)	20 (8%)	37 (6%)	0.33
**CABG**	N (%)	24 (9%)	71 (11%)	0.93
**PCI**	N (%)	31 (12%)	84 (13%)	0.84
**ICD**	N (%)	3 (1%)	19 (3%)	0.17
**Pacemaker**	N (%)	43 (17%)	97 (15%)	0.81
**EF**	Mean (SD)	59.2 (7.7)	58.7 (7.9)	0.73
**EF >50%**	N (%)	236 (91%)	571 (89%)	0.71
**EF >55%**	N (%)	201 (77%)	479 (75%)	0.60
**EF >60%**	N (%)	143 (55%)	317 (50%)	0.21
**EF >65%**	N (%)	69 (27%)	177 (28%)	0.53
**Outcomes**				
**HF hospitalization**	N (%)	41 (16%)	147 (23%)	0.04
**CVD death**	N (%)	33 (13%)	87 (14%)	0.71
**MI**	N (%)	10 (4%)	31 (5%)	0.67
**Stroke**	N (%)	6 (2%)	25 (4%)	0.26
*** Cochran-Mantel-Haenszel chi square test for categorical variables;**				
**conditional logistic regression for continuous variables**				

Baseline characteristics of the propensity score matched cohort of patients that did not change their beta-blocker status during follow-up. ACE-I, Angiotensin Converting Enzyme Inhibitor; ARB, Angiotensin Receptor Blocker; BMI, body mass index; CABG, Coronary Artery Bypass Grafting; CAI, Carbonic anhydrase Inhibitor; COPD, Chronic Obstructive Pulmonary Disease; DBP; diastolic blood pressure; EF, Ejection Fraction; HR, Heart Rate, HTN, Hypertension; ICD, Implantable Cardioverter Defibrillator; K, Potassium; MI, Myocardial Infarction; NYHA, New York Heart Association; PAD, Peripheral Artery Disease; PCI, Percutaneous Coronary Intervention.

© 2019 Silverman DN et al. *JAMA Network Open.*

eTable 4. Beta-blocker Utilization in Contemporary HFpEF Cohorts

Study	Treatment Arms	Major Endpoints	Inclusion Criteria	Mean EF	Baseline beta-blocker use
**CHARM-P**<br>**2003 (n=3,023)**	Candesartan<br>Placebo	CV Mortality<br>HF Hospitalization	LVEF >40%<br>NYHA II-IV	54%	**56%**
**PEP-CHF**<br>**2006 (n=850)**	Perindopril<br>Placebo	All-cause Mortality<br>HF Hospitalization	Clinical DHF	65%	**54%**
**I-PRESERVE**<br>**2008 (n=4,128)**	Irbesartan<br>Placebo	All-cause Mortality<br>CV Hospitalization	LVEF ≥45%<br>NYHA II-IV	60%	**59%**
**RELAX**<br>**2013 (n= 216)**	Sildenafil<br>Placebo	Change in Peak<br>VO_2 on CPET	LVEF ≥50%<br>NYHA II-IV	60%	**76%**
**TOPCAT**<br>**2014 (n=3,445)**	Spironolactone<br>Placebo	CV Mortality, RSD<br>HF Hospitalization	LVEF ≥45%,<br>clinical DHF	56%	**79%**
**NEAT-HFpEF**<br>**2015 (n=110)**	Isosorbide mononitrate<br>Placebo	Exercise Capacity<br>QOL measure	LVEF ≥50%<br>NYHA II-III	63%	**70%**
**INDIE-HFpEF**<br>**2018 (n=105)**	Inorganic nitrate<br>Placebo	Peak Oxygen<br>consumption	LVEF ≥50%,<br>clinical DHF	61%	**63%**
**PARAGON-HF**<br>**2018 (n=4,822)**	Sacubitril/Valsartan<br>Valsartan	CV Mortality<br>HF Hospitalization	LVEF ≥45%<br>NYHA II-IV	58%	**80%**

CHARM-P, Candesartan in Heart Failure Assessment of Reduction in Mortality and Morbidity-Preserved; CPET, cardiopulmonary exercise test; CV, cardiovascular; DHF, diastolic heart failure; HF, heart failure; INDIE-HFpEF, Inorganic Nitrite Delivery to Improve Exercise Capacity in Heart Failure With Preserved Ejection Fraction; I-PRESERVE, Irbesartan in Heart Failure With Preserved Ejection Fraction; EF, left ventricular ejection fraction; NEAT-HFpEF, Nitrate's Effect on Activity Tolerance in Heart Failure with Preserved Ejection Fraction; PARAGON-HF, Prospective Comparison of ARNI with ARB Global Outcomes in HF With Preserved Ejection Fraction; PEP-CHF, Perindopril in Elderly People With Chronic Heart Failure; RSD, resuscitated sudden death; QOL, quality of life

© 2019 Silverman DN et al. *JAMA Network Open.*

eTable 5. Median and Interquartile Ranges for BNP and NT-proBNP Levels

	All EFs			EF <50%			EF ≥50%		
	No Beta-blocker	Beta-Blocker	p-value	No Beta-blocker	Beta-Blocker	p-value	No Beta-blocker	Beta-Blocker	p-value
**BNP**	205 (139-326)	273 (153-475)	0.001	240 (234-298)	364 (249-516)	0.14	198 (138-331)	261 (150-465)	0.002
**NT-proBNP**	889 (621-1694)	972 (554-2148)	0.20	1839 (1380-7333)	967 (526-1630)	0.01	847 (551-1520)	989 (554-2213)	0.03

Median (IQR) of BNP and NT-proBNP by beta-blocker use at baseline in the entire population and stratified by an EF threshold of 50%. P-values compare log-transformed BNP or NT-proBNP values at baseline by beta-blocker use.

© 2019 Silverman DN et al. *JAMA Network Open.*

eTable 6. Hazard Ratios for Cardiovascular Disease Mortality by Ejection Fraction

	**Unadjusted**	**Minimally Adjusted***	**Fully adjusted****
**All EF**	1.08 (0.77, 1.50)	1.11 (0.80, 1.56)	1.16 (0.82, 1.62)
**EF <50%**	0.69 (0.30, 1.58)	0.67 (0.28, 1.62)	0.62 (0.25, 1.58)
**EF ≥50%**	1.14 (0.79, 1.64)	1.21 (0.84, 1.76)	1.24 (0.85, 1.79)

*Adjusted for age, sex, race/ethnicity, and treatment assignment
**Minimally adjusted model plus prior myocardial infarction, atrial fibrillation, chronic obstructive pulmonary disease, asthma, and hypertension

Hazard ratios for cardiovascular disease mortality for beta-blocker use at baseline versus no beta-blocker use at baseline.
.

© 2019 Silverman DN et al. *JAMA Network Open.*

eFigure 1. Cumulative Incidence Plot for Cardiovascular Disease Mortality by Beta-Blocker Use in Patients With an EF ≥50%

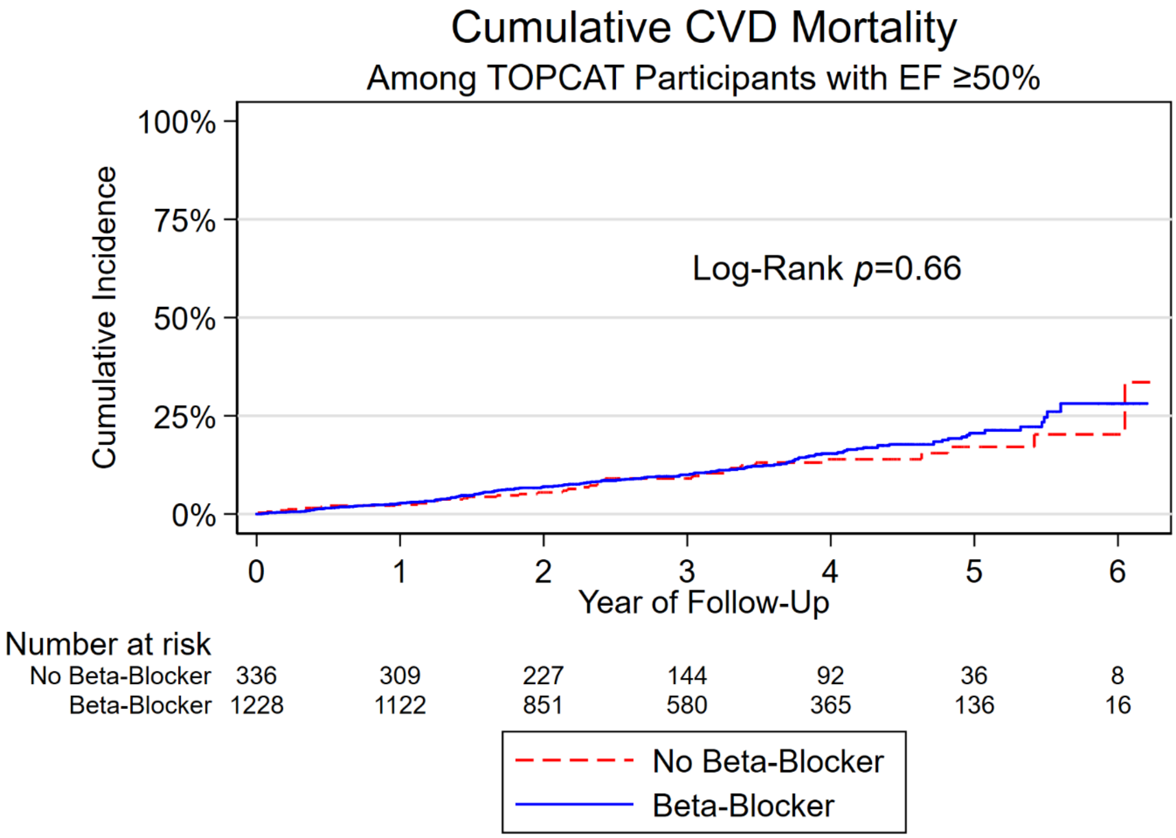

Kaplan Meier plots for CVD mortality by beta-blocker use at baseline stratified by an ejection fraction ≥50%.

© 2019 Silverman DN et al. *JAMA Network Open.*

eFigure 2. Quartile Ranges of BNP and NT-proBNP Levels by Beta-Blocker Use

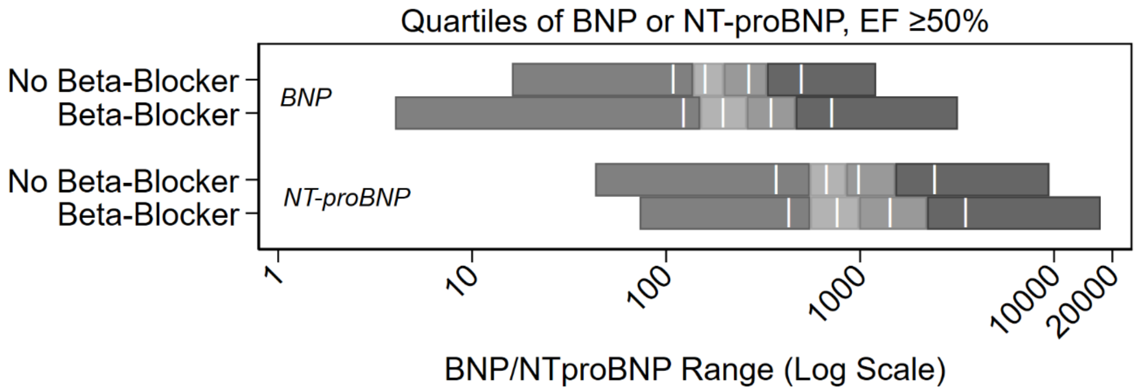

Baseline brain natriuretic peptide (BNP) and N-terminal proBNP (NT-proBNP) levels by beta-blocker at baseline use in patients with an EF ≥50%. Midpoints of the quartiles are indicated by vertical lines.

© 2019 Silverman DN et al. *JAMA Network Open.*
